# Regulation of Vi Capsular Polysaccharide Synthesis in *Salmonella enterica* Serotype Typhi

**DOI:** 10.3855/jidc.154

**Published:** 2008-12-01

**Authors:** Javier M Santander, Kenneth L. Roland, Roy Curtiss

**Affiliations:** Center for Infectious Diseases and Vaccinology, The Biodesign Institute and School of Life Sciences, Arizona State University, Tempe, Arizona 85287-5401, United States of America

**Keywords:** *rpoS*, VW variation, osmolarity

## Abstract

The synthesis of Vi polysaccharide, a major virulence determinant in *Salmonella enterica* serotype Typhi (*S*. Typhi), is under the control of two regulatory systems, *ompR-envZ* and *rscB-rscC*, which respond to changes in osmolarity. Some *S*. Typhi isolates exhibit over-expression of Vi polysaccharide, which masks clinical detection of LPS O-antigen. This variation in Vi polysaccharide and O-antigen display (VW variation) has been observed since the initial studies of *S*. Typhi. We have reported that the status of the *rpoS* gene is responsible for this phenomenon. We review the regulatory network of the Vi polysaccharide, linking osmolarity and RpoS expression. Also, we discuss how this may impact live attenuated *Salmonella* vaccine development.

## Introduction

*Salmonella enterica* serotype Typhi (hereafter *S*. Typhi) is a facultative intracellular pathogen that causes typhoid fever exclusively in humans and is among the most costly of human infections in terms of both morbidity and mortality [1]. The mechanism responsible for the virulence of *S*. Typhi is different from that of other serovars of *Salmonella*, and in this regard *S*. Typhi produces the virulence capsular polysaccharide (Vi), which is an important virulence determinant during infection [2]. Vi polysaccharide is a linear homopolymer made up of α-1,4-linked N-acetylgalactosaminuronate (GalNAcA), with 60%–70% of the monomeric units O-acetylated at the C3 position [3,4] and has a molecular mass typically over 200 kDa [5]. Virtually all strains isolated from the blood or bone marrow of patients with acute typhoid fever and from the bile or faeces of those who carry *S*. Typhi in the gallbladder are found to express Vi polysaccharide antigen [6,7,8]. Vi positive (Vi^+^) strains were shown to be more virulent than $\tilde{\text{Vi}}$ mutant strains in experiments conducted in human volunteers [2]; Vi^+^ strains are resistant to complement-mediated killing and phagocytosis [9] and survive in human serum [10]. In addition, Vi^+^ strains, but not Vi^−^ mutant strains, can multiply in the human macrophage cell line THP-1 and in the mouse macrophage-like cell line J774.1 [11].

The Vi capsular polysaccharide is a protective antigen, with the majority of the antibody response directed toward the O-acetyl groups [9,12]. A Vi-conjugate injectable vaccine is used currently against typhoid fever in more than 92 countries [13]. However, the ability to pay for the conjugate vaccine remains a major factor driving who is and who is not vaccinated. One possible way to increase the supply of affordable Vi vaccine is to optimise the production of Vi during fermentation either by modifying growth conditions [14] or by constructing *S*. Typhi mutants that constitutively express high levels of Vi [15]. Therefore, a better understanding of the regulation of Vi polysaccharide synthesis will not only add to our knowledge base of *S*. Typhi pathogenesis, but also allow us to improve Vi antigen production and therefore provide a less expensive subunit vaccine.

## The *viaB* locus, a part of SPI-7

**Figure 1:**
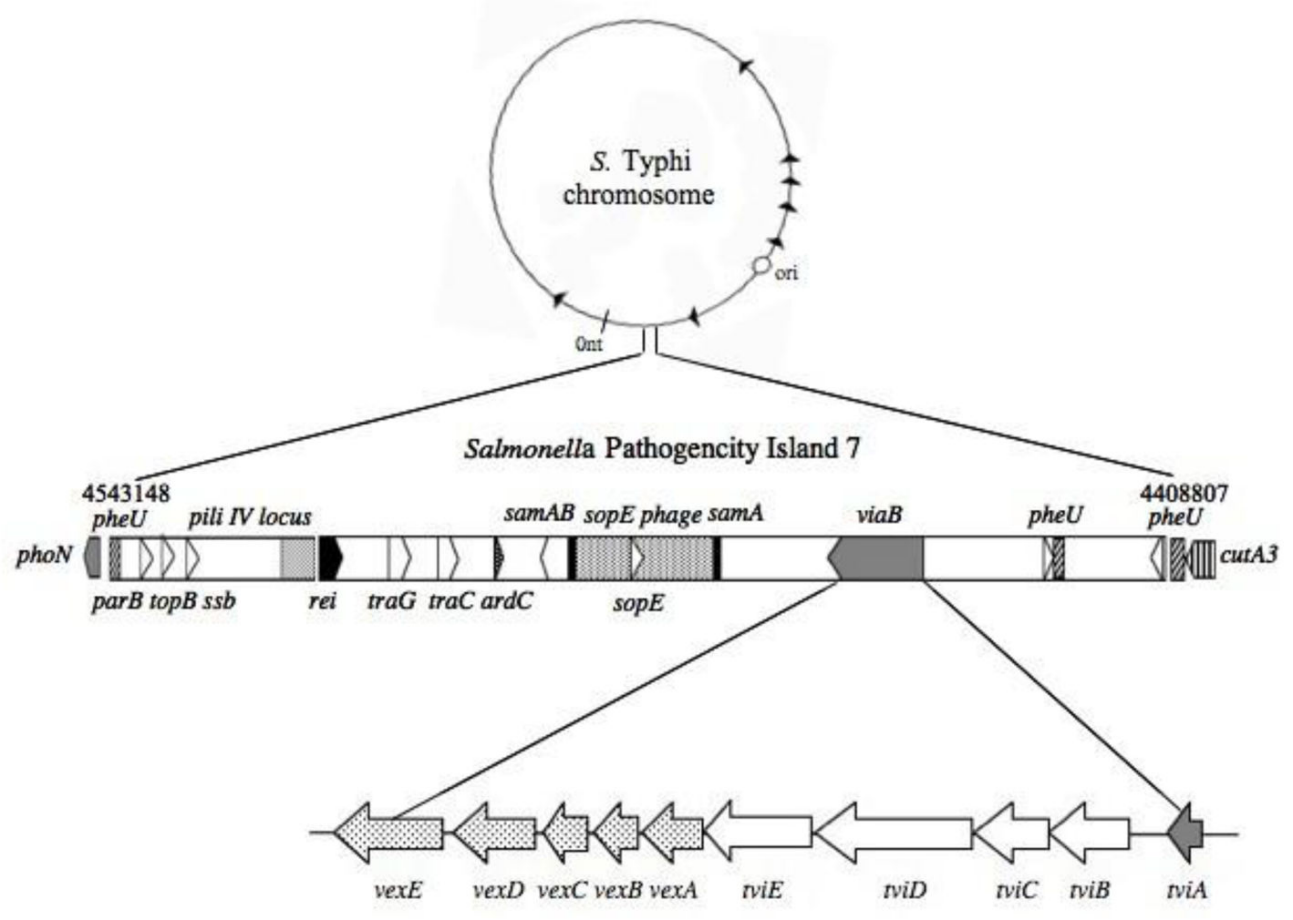
SPI-7 and *viaB* locus organisation in *S*. Typhi. The dark arrows in the *S*. Typhi chromosome indicates the rrn operons. The SPI-7 is located at 96 min or between 4408807 nt and 4543148 nt.

The genes required for Vi biosynthesis (*viaB* locus) are located in a 133.5 kb chromosomal region called *Salmonella* pathogenicity island 7 (SPI-7; G+C composition 49%; Figure 1). SPI-7 is bounded by direct repeats and inserted between two copies (one partial) of a tRNA gene (*pheU*) and contains genes encoding known pathogenicity determinants, including SopE [16] and type IV pili [17,18]. SPI-7 is an unstable genetic element that can undergo precise excision, indicating a possible role in the lateral transfer of this region among Gram negative bacteria [19]. Functional and bioinformatic analyses suggest that SPI-7 has a mosaic structure and may have evolved as a consequence of several independent insertion events [20]. Sequence analysis of the SPI-7 region from *S*. Typhi revealed significant synteny with clusters of genes from a variety of soil saprophytic bacteria and phytobacteria, raising the possibility that SPI-7 and *viaB* may have originated from soil sources [20].

## Biosynthesis of Vi polysaccharide

The *viaB* locus consists of 10 genes (Figure 1): *tviBCDE* for Vi polysaccharide biosynthesis and *vexABCDE* for export of the Vi antigen [10, 21, 22], as well as *tviA*, which encodes a regulatory protein that plays a role in coordinating expression of Vi antigen, flagella and a number of genes required for host invasion [23,24,25,26]. Zhang *et al*. have provided a detailed analysis of Vi biosynthesis [5]. They showed that Vi polysaccharide is synthesised from UDP-N-acetylglucosamine in a series of steps requiring TviB, TviC, and TviE, where *tviB* encodes a dehydrogenase, *tviC* encodes an epimerase and *tviE* encodes a glycosyltransferase [5]. The role of *tviD* is not clear, but it appears to encode a cytochrome P-450-like enzyme [5].

## Export of Vi polysaccharide

The Vi antigen export apparatus is composed of five polypeptides: VexA, VexB, VexC and VexD, which likely form an ABC transporter, and the VexE anchoring protein [22,27,28]. Mutants defective in VexA, VexB and VexC accumulate the Vi polysaccharide in their cytoplasm [29]. VexB and VexD have been proposed to be integral membrane proteins because they are highly hydrophobic proteins with membrane-spanning domains [27,29]. VexC is likely the ATP-binding protein [27]. VexA has a putative lipoprotein signal sequence indicating that this protein may be localised in the outer membrane and could therefore mediate the translocation of Vi polysaccharide across the outer membrane [22,25,30]. *S*. Typhi Ty2 *vexE* mutants are able to synthesise and export the Vi antigen, but do not express it on its cell surface [29]. Thus VexE is necessary for cell surface expression of the Vi capsule.

## Regulation of Vi polysaccharide synthesis

**Figure 2:**
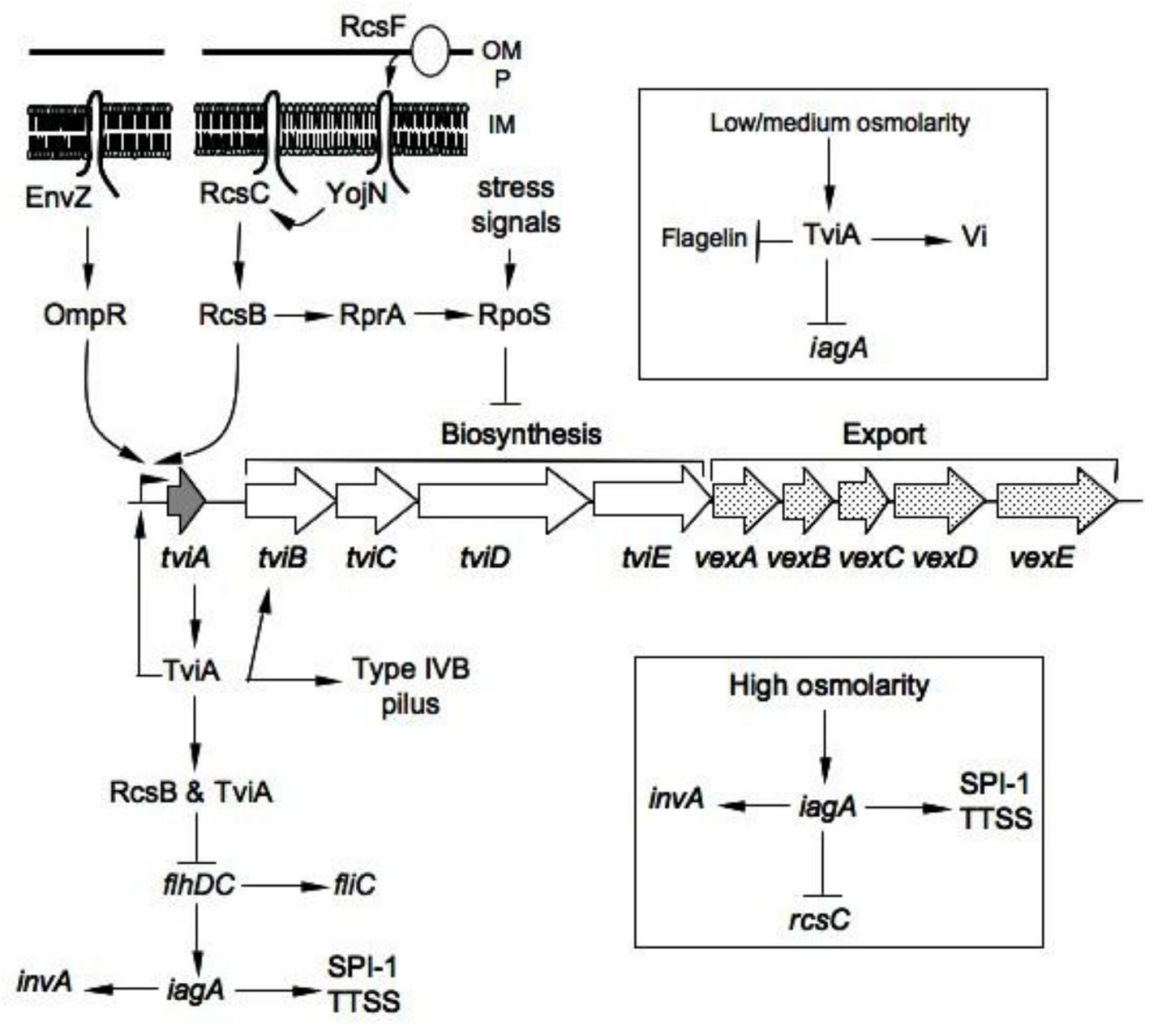
Regulatory network of Vi polysaccharide. Two two-component regulatory systems are involved in the regulation of Vi antigen expression in *S*. Typhi. The *rcs* system positively regulates transcription of *tvi* genes. Moreover, interaction between TviA and RcsB proteins is necessary for maximal transcription of *tvi* genes. OmpR/EnvZ is the second regulatory pair involved in the regulation of Vi polysaccharide expression. An increase in the environmental osmolarity leads to negative regulation of Vi antigen synthesis by inhibition of *rcsC* transcription. Molecular mechanisms involved in this regulation remain to be determined. OM: Outer membrane; P: Periplasm; IM: Inner membrane.

The regulation of Vi polysaccharide synthesis is complex and may play a significant role in maintaining appropriate levels of Vi production in the different host environments encountered by *S*. Typhi. In Figure 2, we have outlined our current understanding of this regulation. Vi antigen synthesis is subject to regulation by a pair of two-component systems, *rcsB-rcsC* (*viaA* locus) [25] and *ompR-envZ* (*ompB* locus) [26], which both respond to changes in osmolarity. A positive regulator, TviA (VipR), activates its own synthesis by binding upstream of the *tviA* promoter [27] and interacts with RcsB to promote optimal transcription of genes involved in Vi antigen synthesis [24, 25, 28, 29]. In the absence of RcsB or TviA, transcription initiated at the *tviA* promoter terminates in the *tviA-tviB* intergenic region, probably at a putative hairpin structure identified in this region [29].

Vi is expressed preferentially at low and medium osmolarities and often masks the LPS O-antigen [25,31]. Strains of *S*. Typhi Ty2 grown in media with medium osmolarity (446 mosmol, ~170 mM NaCl [31]) exhibit high-level production of Vi antigen. When Vi antigen is expressed, the bacteria are less adherent and invasive into epithelial cells [30] but are more resistant to killing by macrophages [11]. Low to medium osmolarity environments might include environmental aqueous environments and certain extracellular host environments, such as blood, where the osmolarity is equivalent to 150 mM of NaCl (310 mosmol) [31,32]. It is possible that this preferential expression of Vi polysaccharide at low to medium osmolarities serves to protect bacterial cells from the complement-mediated actions of the O-antigen specific antibody in the blood [9]. Recent studies with *S*. Typhi Ty2 grown under LB conditions (170 mM NaCl), optimal for Vi polysaccharide synthesis, showed that Vi polysaccharide reduced TLR-dependent IL-8 production in human colonic tissue explants, suggesting that the scarcity of neutrophils in intestinal infiltrations of typhoid fever patients is due to the Vi polysaccharide [33]. In addition, at low osmolarity, RcsB, acting in association with TviA, negatively controls the transcription of *flhDC*, which is apparently required for activation of *iagA* (*hilA*), i*nvF* and *sipB* (encoding proteins involved in cell invasion) [25, 34]. However, in high osmolarity environments such as in the intestinal lumen, with values believed to be equivalent to 300 mM of NaCl and greater [31], transcription of *iagA*, *invF*, and *sipB* is markedly increased and the transcription of genes involved in Vi biosynthesis is markedly decreased [25]. Under these conditions, *S*. Typhi is more invasive into epithelial cells but less resistant to killing by macrophages [11]. Therefore, the Vi antigen of *S*. Typhi is a negative factor for invasion but a positive factor for surviving and multiplying inside macrophages [11]. In keeping with this observation, Zhao *et al*. showed that at ≥ 300 mM NaCl, but not at 10 mM NaCl, *S*. Typhi GIFU1007 did not express Vi antigen and exhibited a high invasion index in epithelial cells together with high secretion of SipC protein [35].

## VW variation a subtle regulation of Vi polysaccharide by RpoS

**Figure 3:**
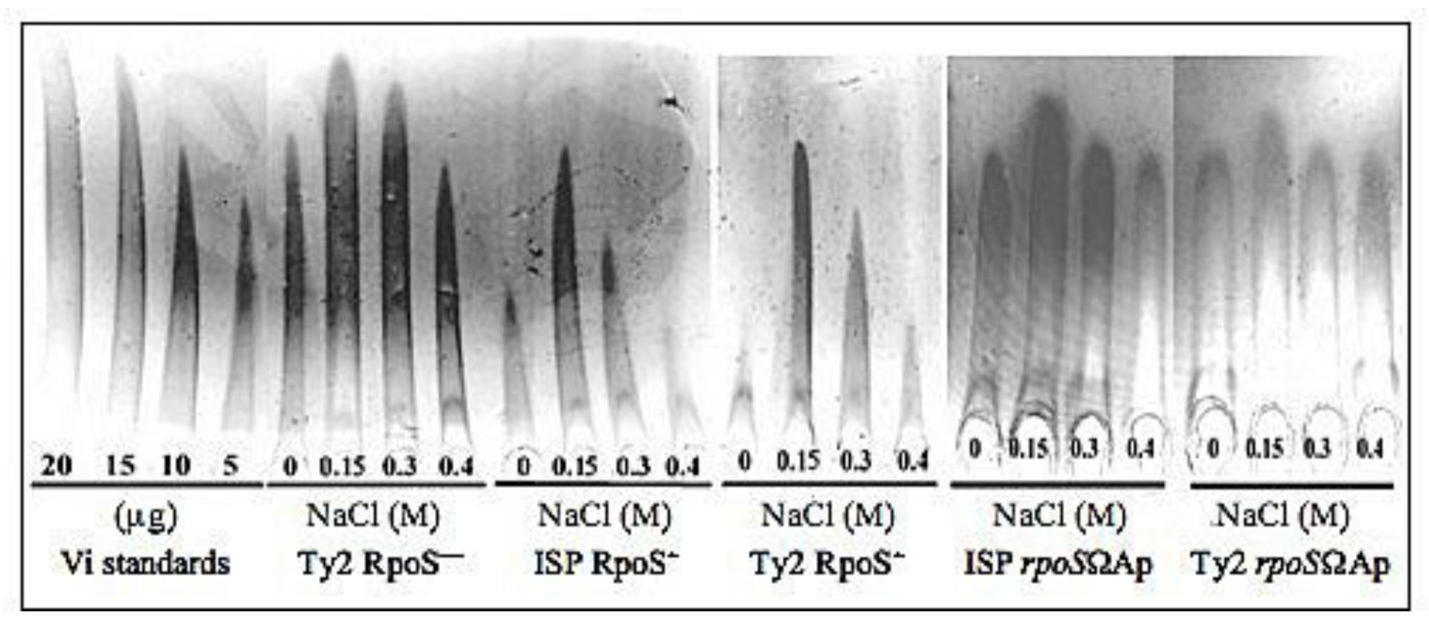
Evaluation of the effect of RpoS in the synthesis of Vi polysaccharide in *S*. Typhi by rocket immune electrophoresis. *S*. Typhi Ty2 RpoS^−^; *S.* Typhi ISP1820 RpoS^+^; *S*. Typhi Ty2 RpoS^+^ dervivative; *S*. Typhi Ty2 *rpoS*ΩAp (RpoS^−^) derivative; *S*. Typhi ISP1820 *rpoS*ΩAp (RpoS^−^) derivative; The strains were grown in LB media with 0, 0.15, 0.3 and 0.4 M NaCl. Reproduced with permission from Santander *et al*. [51].

The synthesis of Vi antigen increases as the osmolarity decreases (Figure 3), masking the O antigen (Table 1 and [24]). At osmolarities of 676 mosmol (300 mM of NaCl) or higher, the Vi antigen no longer blocks detection of the O-antigen (Table 1). Since the Vi polysaccharide can block access of antibodies to the underlying O-antigen, sometimes agglutination with *Salmonella* somatic D1 antiserum cannot be demonstrated until the bacterial cells are boiled to remove the Vi polysaccharide [36].

Starting from the initial studies on *S*. Typhi, variation in Vi and O-antigen detection has been observed. *S*. Typhi strains non-agglutinable with O-antisera and agglutinable only with Vi antisera, are called V form while *S*. Typhi strains that lack the Vi antigen and agglutinate only with O-antisera are called W form [37]. Observations recorded by Kauffman [38] and confirmed by Felix and Pitt [39] demonstrated the concept of VW variation in Vi and O antigen relationships. The VW form, which is the most common form observed in clinical laboratories, is defined when both Vi and O-antigen are detected by agglutination with the respective antisera [37]. Coincident with the early work of Felix and Pitt [39], and since verified by others [11,40,41,42], most virulent strains of *S*. Typhi, were the VW form.

### Effect of *rpoS* on Vi synthesis

In *Salmonella*, the *rpoS* gene encodes an alternative sigma factor (σ^s^/RpoS) that is the master regulator in the general stress response and is required for survival under extreme conditions, including osmotic and oxidative stress, transition to stationary phase, acid shock, [43,44] and for virulence of *S*. Typhimurium [45,46,47, 48,49]. RpoS controls expression of the *S*. Typhimurium virulence plasmid genes, *spvRABCD* [46, 47]. In addition, RpoS regulates chromosomal genes required for colonisation of Peyer’s patches and for persistence in mice [48,49]. *S*. Typhi does not contain a virulence plasmid and the role of *rpoS* in the virulence of this serotype has not been rigorously studied. However, *rpoS* might also contribute to the virulence of this serotype because RpoS^−^ strains of *S*. Typhi are less cytotoxic than RpoS^+^ strains, but RpoS^−^ strains survive better inside resting THP-1 macrophages without apoptosis induction [50]. Recent studies in our laboratory indicate that there is a correlation between Vi polysaccharide over-expression and the allelic state of the *rpoS* gene [51].

High osmolarity is one of the environmental signals that induces *rpoS* through the RcsB-RprA pathway [43,48]. Since the osmolarity of the growth media influences the synthesis of Vi antigen, we examined strains with different *rpoS* genotypes, in media with different osmolarities, for levels of Vi antigen synthesis. RpoS^+^ strains grown at osmolarities less than 676 mosmol (~300 mM NaCl [31]) showed over-expression of Vi antigen sufficient to cover the somatic O-antigen (Table 1). The phenotypically O-antigen negative strains were boiled and the O-antigen was subsequently detected in all cases, indicating that Vi was masking the intact LPS. The level of Vi polysaccharide synthesis was higher in RpoS^−^ strains than in RpoS^+^ strains, although Vi synthesis was responsive to changes in osmolarity for both genotypes (Table 1; Figure 3). Pickard *et al*. [24] showed in *S*. Typhi vaccine strains that the *ompB* locus is required for Vi synthesis and is influenced by osmolarity. These authors also observed that *S*. Typhi Ty2 and *S*. Typhi ISP1820 have different levels of Vi antigen synthesis when these strains were grown in different osmolarities. They suggested that this difference could be due to a mutation in Ty2 owing to *in vitro* passage, since it is an older isolate. In fact, it was subsequently shown that *S*. Typhi Ty2 carries an *rpoS* frame-shift mutation [52]. However, it is not clear whether this mutation was present in the original isolate or is a result of laboratory passage. In one recent study, 36% of fresh human *S*. Typhi isolates were found to be *rpoS* mutants [53]. We confirmed that the *rpoS* allelic state is in fact responsible for these observations by constructing an *rpoS* deletion in ISP1820 [51]. In addition, we constructed an RpoS^+^ derivative of Ty2. RpoS^+^ Ty2 had the same Vi phenotype as ISP1820 and the RpoS^−^ derivative of ISP1820 had the same phenotype as Ty2 (Table 1).

Rocket immune electrophoresis assays indicated that RpoS^+^ strains down-regulate Vi antigen expression (Figure 3). Maximum Vi polysaccharide levels were observed at 150 mM NaCl for both Ty2 (RpoS^−^) and ISP1820 (RpoS^+^), but Ty2 produced more Vi than ISP1820 at all osmolarities tested (Figure 3). These results support our interpretation of the agglutination results in Table 1 and indicate that RpoS down-regulates Vi polysaccharide synthesis in *S*. Typhi. The molecular mechanism governing how and why RpoS accomplishes this is still an open question.

### Effect of *rpoS* on Vi antigen synthesis and the effect on H_d_ detection

**Figure 4:**
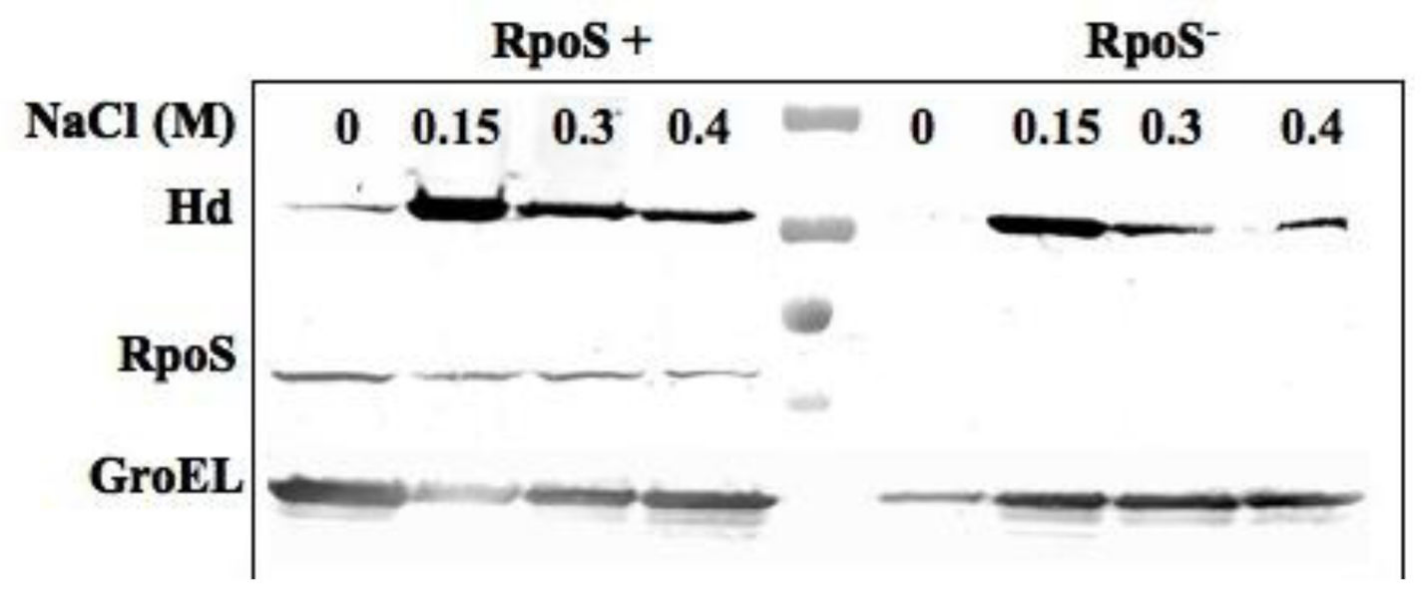
Evaluation of H_d_ (*S*. Typhi flagella factor; 55 kDa) and RpoS expression in different osmolarities by western blot. *S*. Typhi Ty2 RpoS^−^; *S*. Typhi ISP1820 RpoS^+^; The strains were growth in LB media with 0, 0.15, 0.3 and 0.4 M of NaCl. Reproduced with permission from Santander *et al*. [51].

The allelic variant of the *fliC* gene present in *S*. Typhi strains encodes one type of flagellin, designated H_d_ [25]. *S*. Typhi GIFU10007 appeared to require intrinsic, intact motility for invading cultured epithelial cells, as non-motile mutants were not invasive [54]. The production of the H_d_ flagellin, as well as Vi antigen, is modulated by the RcsB-RcsC regulatory system in response to changes in the osmolarity of the growth medium [25]. We have examined H_d_ synthesis in strains with different *rpoS* genotypes grown in media with different osmolarities [51]. The synthesis of H_d_ flagellar antigen in RpoS^+^ strains was detected at all osmolarities tested. In contrast, the H_d_ flagellar antigen was not detected in RpoS strains at osmolarities of 10 mM NaCl or lower (Table 2) unless the samples were first boiled, indicating that the Vi antigen masks flagellar antigens at low osmolarities in RpoS strains. Western blot analysis revealed a low level of H_d_ flagellin synthesis when cells were grown in a low osmolarity medium, as expected (Figure 4). Similar results were obtained using arabinose inducible *rpoS* strains [51], where masking of flagellar antigens at low osmolarity was only observed when cells were grown in the absence of arabinose [51].

### Evaluation of Vi polysaccharide synthesis in *S*. Typhi RpoS^+^ strains during growth

**Figure 5:**
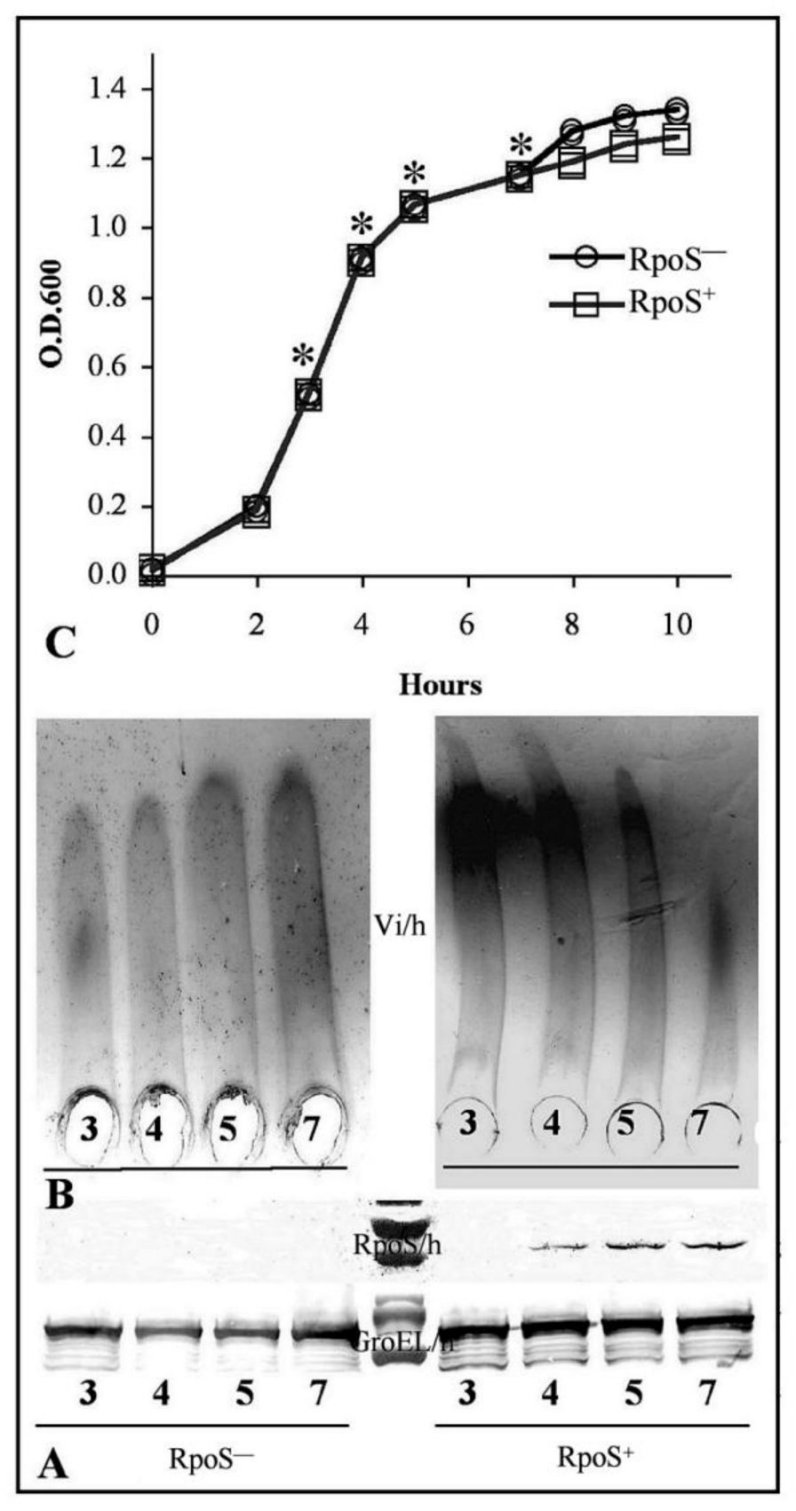
Evaluation of Vi polysaccharide synthesis in *S*. Typhi RpoS^+^ during growth. A. RpoS expression during the growth curve. GroEL was used as control. The strains were growth in LB medium with 150 mM of NaCl; B. Vi polysaccharide expression during the growth; C. Growth curve. *S*. Typhi Ty2 RpoS^−^; *S*. Typhi ISP1820 RpoS^+^. Reproduced with permission from Santander *et al*. [51].

RpoS is a key factor in the stress response during the transition from the exponential growth phase to the stationary growth phase [43]. Very little RpoS is detected in exponentially growing *E. coli* cells, due to either low levels of expression or protein instability [56,57]. To determine what impact this might have on Vi expression, we evaluated the effect of growth phase on Vi and RpoS synthesis in *S*. Typhi. RpoS was not detected in the early exponential phase, but was detectable from the middle exponential growth phase cultures and into the early stationary phase (Figure 5), although bubble production in the catalase test was positive only in the stationary phase. Notably, Vi polysaccharide synthesis decreased as RpoS accumulated (Figure 5). There was no growth phase dependent reduction in Vi antigen synthesis in strain Ty2 (RpoS^−^). These results indicate that the RpoS allelic state is responsible for the VW and V variation in *S*. Typhi. RpoS^−^ strains over-express the Vi polysaccharide without RpoS regulation, leading to a permanent V form. RpoS^+^ strains exhibit both forms, the V form during the early exponential growth, when RpoS is not expressed or expressed at low levels, and the VW form, when RpoS is expressed. The W form (Vi^−^) can be caused by spontaneous deletion of SPI-7, where the *viaB* loci is located [19].

## Vaccine development

The live typhoid vaccine Ty21a, which is an RpoS, GalE^−^ and Vi^−^ [57] derivative of *S*. Typhi Ty2 [58], has been evaluated in several clinical trials and found to be well tolerated, although only modesty immunogenic; three or four doses are required to confer protection [59,60,61,62]. The *rpoS* mutation could affect the immunogenicity in recombinant vaccines [63]. In fact, live typhoid vaccines derived from Ty2 (RpoS^−^) have yielded poor results when used as a live recombinant *S*. Typhi vaccine (RAStyVs) expressing protective antigens from a diversity of pathogens [64,65,66,67,68,69]. It is thus possible that the poor immunogenicity observed for Ty21a may be the result of the *rpoS* mutation rather than Vi antigen deletion. On the other hand, over-expression of Vi antigen in Ty2 could decrease adherence to and invasion into intestinal tissues necessary to colonise more internal lymphoid effector tissues [64]. In *S*. Typhimurium, it has been demonstrated that chromosomal RpoS-regulated genes are necessary for invasion into and colonisation of the gut-associated lymphoid tissue (GALT) [49]. In accord with this, RpoS^−^ *S*. Typhimurium mutants exhibit diminished immunogenicity [70,71]. The RpoS-regulated genes carried on the *S*. Typhimurium virulence plasmid appear to play no role in this effect [70,71].

Furthermore, *S*. Typhi ISP1820 (RpoS^+^) seems to be more virulent in humans than *S*. Typhi Ty2 (RpoS^−^) [72]. In concordance, *S*. Typhi CVD906, a live vaccine, derived from ISP1820 with deletion mutations caused fever and other adverse reactions in humans in *aroC* and *aroD* [73], is highly immunogenic but [72,74]. This is in contrast to the *S*. Typhi CVD908 Ty2 Δ*aroC* Δ*aroD* vaccine strain, which did not cause any adverse effects [72]. These results collectively imply that RpoS^+^ *S*. Typhi, with or without ability to produce the Vi capsular antigen, might be superior to RpoS^−^ strains as a vector in the development of recombinant attenuated *Salmonella* vaccines for humans [75,76], although they will require more effective means of attenuation that has been used previously in *S*. Typhi Ty2 vaccine constructions. We are currently testing this hypothesis in human volunteers.

## Figures and Tables

**Table 1:** O9 and Vi slide agglutination reactions of *S*. Typhi strains grown on LB agar (pH 7) supplemented with different amounts of NaCl at 37°C overnight (18–24 h).

	Environment				Blood-Fluid tissues			

NaCl (mM)	0		10		85		150	

Strains	O9	Vi	O9	Vi	O9	Vi	O9	Vi
Ty2 RpoS−	−	+++	−	+++	−	+++	−	+++
ISP RpoS+	++	++	+++	++	++	++	++	++
Ty2 RpoS+	++	++	+++	++	++	++	+++	++
Ty2 *rpoS*ΩAp	−	+++	−	+++	−	+++	−	+++
ISP *rpoS*ΩAp	−	+++	−	+++	−	+++	−	+++

	Intestinal lumen					

NaCl (mM)	300		400		500	

Strains	O9	Vi	O9	Vi	O9	Vi
Ty2 RpoS−	++	+++	+++	+	+++	−
ISP RpoS+	+++	−	+++	−	+++	−
Ty2 RpoS+	+++	−	+++	−	+++	−
Ty2 *rpoS*ΩAp	++	+++	+++	+	+++	−
ISP *rpoS*ΩAp	++	+++	+++	+	+++	−

O_9_ agglutination reactions were carried out without prior boiling of cells. The degree of agglutinations ranged from not detectable (−) to weak (+) to strong (+++); ± and ++ indicate intermediate degrees. Adapted with permission from Santander *et al*. [51].

**Table 2:** H_d_ flagellar antigen slide agglutination reactions of *S.* Typhi strains grown on LB agar (pH 7) [76] supplemented with different amounts of NaCl at 37°C overnight (18–24 h).

Strains	NaCl (mM)						
	0	10	85	f	300	400	500
Ty2 RpoS^−^	−	−	+++	+++	+++	+++	+++
ISP RpoS^+^	++	+++	+++	+++	+++	+++	+++
Ty2 RpoS^+^	++	+++	+++	+++	+++	+++	+++
Ty2 *rpoS*Ω Ap	−	−	+++	+++	+++	+++	+++
ISP *rpoS*Ω Ap	−	−	+++	+++	+++	+++	+++

The degree of agglutinations ranged from not detectable (−) to weak (+) to strong (+++);± and ++ indicate intermediate degrees. Adapted with permission from Santander et al. [51]
